# Unmasking Cerebral Venous Sinus Thrombosis Induced by Vitamin B12 Deficiency

**DOI:** 10.7759/cureus.94635

**Published:** 2025-10-15

**Authors:** Danielle Pitter, Daniel M Schachter

**Affiliations:** 1 Neurology, Emory University School of Medicine, Atlanta, USA

**Keywords:** aphasia, cerebral venous sinus thrombosis (cvst), hyperhomocysteinemia (hhcy), mr venography, serum vitamin b12

## Abstract

This is the case of a healthy, 45-year-old right-handed man with no past medical issues who presented with mild drowsiness, language difficulty, right-sided weakness, and headache. Vital signs were unremarkable, and on examination, he was drowsy and found to have expressive aphasia and right upper and lower extremity flaccid paralysis. The patient was found to have cerebral venous thrombosis on imaging, as a result of his vitamin B12 deficiency from a vegan diet. The purpose of this clinical reasoning case is to instruct readers on the localization of symptoms, key imaging findings, approach to the diagnosis and etiologic considerations, and the relevance of biochemistry to the pathophysiology of this case.

## Introduction

The cerebral venous system is composed of the cerebral veins and the dural venous sinuses, which is further characterized by superficial and deep systems [1]. The superficial venous system drains from the cortex, while the deep venous system drains from the deep brain structures [1]. Cerebral venous sinus thrombosis (CVST) occurs when there is an occlusion of a venous sinus and subsequent hydrostatic pressure [2]. CVST characteristically presents with headaches, seizures, and focal neurological deficits [2,3]. CVST presents similarly to strokes but only accounts for about 0.5% of them [4]. CT or MR venography is the diagnostic study of choice [2,3]. When analyzing these images for CVST, a filling defect can be noted in the venous sinuses [4]. Some pathognomonic findings include the empty triangle sign, empty delta sign, cord sign, etc. [4]. CVST is commonly treated with anticoagulants, such as warfarin or heparin [1,4,5]. Multiple factors increase the risk of CVST; however, an underrecognized risk factor includes B12 deficiency [2-4]. Due to the role of B12 in homocysteine metabolism, the lack of B12 increases homocysteine levels inducing a prothrombotic state.

## Case presentation

A 45-year-old right-handed man with no past medical history presented to the emergency department with mild drowsiness, expressive aphasia, right upper extremity (RUE) weakness, right lower extremity (RLE) weakness, right toe twitching, and worsening holocephalic headaches for one week. His symptoms began with worsening headache, nausea, and vomiting. A week after the onset of headache and nausea, he developed weakness in the RUE and RLE, at which point he sought medical attention. His family history was notable for paternal prostate cancer. The patient followed a vegan diet, worked as a truck driver, and reported marijuana use. 

Vital signs were within normal limits upon arrival. The initial neurologic examination showed that he was drowsy but arousable with verbal stimulus. Expressive language output was diminished, out of proportion to alertness. He followed simple commands, answered yes/no questions, and had intact cranial nerves. He had flaccid paralysis in the RUE and 2/5 strength in the RLE. Sensation to light touch was intact. Tendon jerk reflexes were 1+ throughout. Plantar reflexes were equivocal. Coordination and gait were non-contributory, in proportion to the level of weakness. 

Complete blood count (CBC), comprehensive metabolic panel (CMP), hemoglobin A1c (A1c), thyroid-stimulating hormone (TSH), lipid panel, HIV, and rapid plasma reagin (RPR) are routinely collected in patients with stroke-like symptoms. The patient had a normal A1c, normal TSH, low LDL, negative HIV, negative SARS-COV-2 PCR, positive treponemal IgG EIA, non-reactive RPR, an unremarkable CBC/CMP, and an elevated PT/INR and PTT (Table 1). Additional labs showed elevated lactate dehydrogenase, normal folate, low Vitamin B12, negative intrinsic factor antibodies, elevated methylmalonic acid, and elevated homocysteine level (Table 1). Protein C, S, and antithrombin were all within normal limits (Table 1). The patient’s labs were negative for lupus anticoagulant, anti-cardiolipin, B2 glycoprotein, and MTHFR mutation. In this case, the patient’s vegan diet likely led to low B12 levels. A lack of B12 leads to elevated levels of homocysteine and methylmalonic acid.

**Table 1:** Summary of quantitative lab values.

Test	Result	Reference range
Labs		
White blood cells	6.2 K/mcL	3.8-10.7 K/mcL
Hemoglobin	10.7 g/dL	13.2-17.7 g/dL
Hematocrit	33.4%	40.3-53.1%
Platelets	237 K/mcL	148-362 K/mcL
Sodium	135 meq/L	132-144 meq/L
Potassium	4.4 meq/L	3.4-5.1 meq/L
Chloride	101 meq/L	101-111 meq/L
CO_2_	24 meq/L	22-32 meq/L
Anion gap	10	1-13
Glucose	109 mg/dL	70-125 mg/dL
Blood urea nitrogen	12 mg/dL	8-22 mg/dL
Creatinine	0.7 mg/dL	0.7-1.2 mg/dL
Prothrombin time	13.4 s	9.4-12.5 s
International normalized ratio	1.2	0.9-1
Partial thromboplastin time	23.4 s	25.1-36.5 s
Lactate dehydrogenase	206 U/L	91-180 U/L
Folate	10.1 ng/mL	>=7.8 ng/mL
Vitamin B12	56 pg/mL	180-914 pg/mL
Methylmalonic acid	2057 nmol/L	55-335 nmol/L
Homocysteine	77.9 umol/L	<11.4 umol/L
Hemoglobin A1c	5.3%	4-5.6%
Low-density lipoprotein	86 mg/dL	100-129 mg/dL
Thyroid stimulating hormone	0.46 uIU/mL	0.34-5.60 uIU/mL
Protein C	117%	60-140%
Protein S	110.9%	63-149%
Antithrombin	114%	80-120%

**Figure 1:**
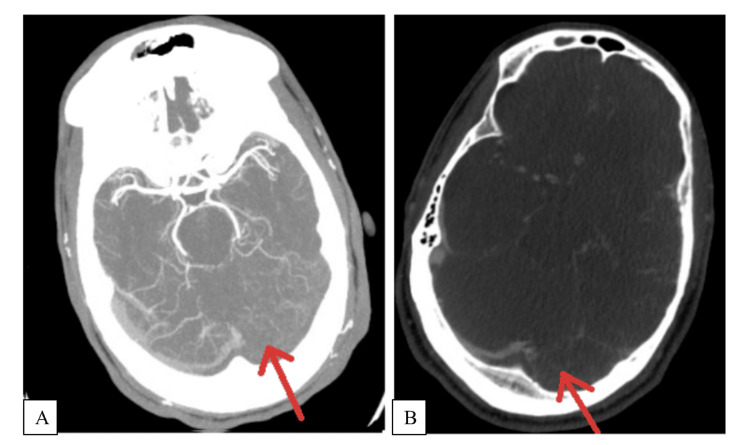
CTA and CTV showing CVST. (A) CTA head and neck showing absent contrast opacification of the left transverse sinus (arrow). (B) CTV showing dural venous sinus thrombosis involving the left transverse sinus (arrow). CTA: CT angiography; CTV: CT venogram; CVST: cerebral venous sinus thrombosis.

Cranial computerized tomography was unremarkable, and CT angiography (CTA) of the head and neck showed absent contrast opacification of the superior sagittal, straight, left transverse, and sigmoid sinuses (Figure 1A). CT venogram (CTV) demonstrated extensive dural venous sinus thrombosis involving the superior sagittal, bilateral transverse, left sigmoid sinus, and superior jugular vein with cortical venous thrombosis involving bilateral frontal and left parietal cortical veins (Figure 1B).

**Figure 2:**
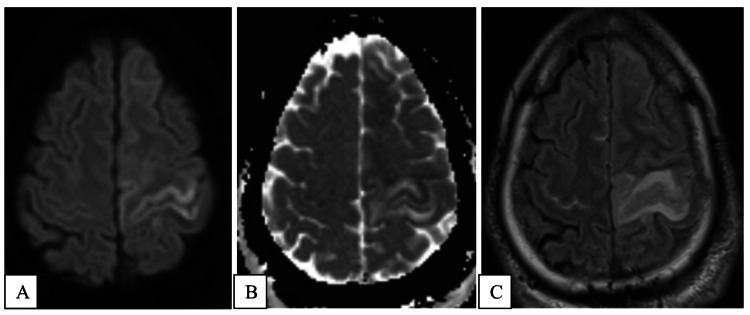
MRI sequences showing restricted diffusion in precentral gyrus. (A) DWI, (B) ADC, and (C) T2 Flair sequences showing restricted diffusion/hyperintense signal in a gyriform pattern spanning the precentral gyrus. DWI: diffusion-weighted imaging; ADC: apparent diffusion coefficient; MRI: magnetic resonance imaging.

Cerebral magnetic resonance imaging brain with and without contrast corroborated findings of CVST, as well as bilateral smaller cortical vein thrombosis. Diffusion-weighted imaging (DWI) and apparent diffusion coefficient (ADC) sequences demonstrated restricted diffusion in a gyriform pattern spanning the left precentral gyrus (Figure 2A and B). T2 Flair sequence showed hyperintense signal spanning the precentral gyrus, which involved a larger volume of brain parenchyma than the corresponding areas of restricted diffusion (Figure 2C). There was also T2 hyperintensity in few bilateral subarachnoid spaces, indicating petechial subarachnoid hemorrhage. EEG showed continuous left temporal slowing but no evidence of seizure activity.

This patient was treated first with IV heparin drip and serial CT monitoring, then with Lovenox 80 mg twice daily, and was transitioned to Eliquis 5 mg twice daily. The etiology of the CVST in this patient was highly suspected to be related to his hyperhomocysteinemia. He was given an intramuscular dose of 1000 mcg of intramuscular B12 and opted to take an oral supplement after discharge. Due to the patient’s history of potential exposure and the positive treponemal IgG EIA, he was treated for late latent syphilis with three weekly injections of IM PCN G 2.4 million units. The patient was scheduled for a neurology appointment two months later but was lost to follow-up.

## Discussion

Differential diagnosis for weakness and holocephalic headache includes primary neurological, rheumatological, immunological, infectious, endocrinological, or genetic causes [6], such as stroke, transient ischemic attack (TIA), CVST, Todd's paralysis, a brain mass, or infection. The unilateral weakness helps confirm central nervous system involvement [6]. The constellation of language and unilateral motor impairment localizes the lesion to the cortex and underlying white matter [7]. The restricted diffusion and corresponding low ADC signal on MRI along the motor strip indicate the area of infarct. The surrounding T2 hyperintense areas in the absence of restricted diffusion represent areas of edema. Venous infarcts from CVST have a high likelihood of forming surrounding vasogenic edema due to hydrostatic pressure caused by venous stasis.

While the patient’s employment as a truck driver is a risk factor for lower extremity deep vein thrombosis, immobilization does not affect the coagulability of cerebral venous blood. Bilateral lower extremity dopplers were unremarkable. One etiologic consideration for anyone with a seemingly unprovoked deep vein thrombosis is malignancy. CT chest, abdomen, and pelvis with IV contrast were negative for malignancy. Additional etiologic work-up for stroke in the young includes genetic and acquired hypercoagulability panels, as well as an infectious workup. Although this patient tested negative for SARS-COV-2, this virus has been associated with CVST in patients by causing endothelial dysfunction and hypercoagulability [8].

In this case, the patient’s vegan diet and vitamin B12 deficiency were the underlying cause of CVST. Vitamin B12 is a cofactor in the biochemical conversion of the amino acid homocysteine to methionine and methylmalonyl-CoA to succinyl-CoA [9,10]. The deficiency of B12 leads to elevated levels of methylmalonic acid and homocysteine. The elevated levels of homocysteine has been found to induce a prothrombotic state in the body through various mechanisms. Hyperhomocysteinemia can lead to increases in oxidative stress and upregulation of inflammatory mediators, which in turn induce endothelial dysfunction [11]. Hyperhomocysteinemia also reduces the efficacy of antithrombin III by inhibiting its binding capabilities to the vessel wall endothelium [12]. Hyperhomocysteinemia leads to numerous downstream effects, which result in a prothrombotic state, such as platelet aggregation and the inactivation of protein C [12]. These mechanisms explain how hyperhomocysteinemia leads to a prothrombotic and inflammatory state, ultimately inducing thrombosis in the body. This case emphasizes the importance of monitoring vitamin levels and considering vitamin deficiencies as an etiology in patients presenting with CVST.

## Conclusions

In conclusion, the presence of elevated homocysteine levels was determined to be the etiology of this patient’s CVST and, fortunately, represented a correctable risk factor for thrombotic events. Symptomatic CVST, depending on the extent of parenchymal infarct, size, and presence of ICH, is treated with anticoagulation. Although it is well known that hyperhomocysteinemia and vitamin B12 deficiency can be associated with CVST, this case is unique because it highlights the presence of a CVST arising solely from a vegan diet, without evidence of a genetic/autoimmune etiology. This case emphasizes the importance of gathering a thorough history, including diet history, as that can offer clues into a patient's presentation. Diabetes, hypertension, and atherosclerosis are very common causes of thrombosis, but other etiologies are to be considered, such as vitamin deficiencies.
